# A Retrospective Case Series of Stenotrophomonas maltophilia at an Acute Care Hospital in Alabama, July-November 2023

**DOI:** 10.1017/ash.2024.263

**Published:** 2024-09-16

**Authors:** Kate Draper, LyTasha Crum, Devon Sims, Melanie Roderick, L. Amanda Ingram

**Affiliations:** Alabama Department of Public Health; Jefferson County Department of Health

## Abstract

**Background:** In August 2023, an infection preventionist at an acute care hospital in Alabama recognized an increase in cases of Stenotrophomonas maltophilia, which is an emerging pathogen in clinical settings worldwide. It was not until the facility identified the pathogen in their water system in October that it was reported to ADPH as an outbreak. The outbreak investigation was brief due to the hospital’s rapid containment response and adherence to its established water management program (WMP). As a result of inappropriate antibiotic use in hospitals, pan-resistant strains have been increasing at an alarming rate. The pathogen can employ water used in hospital settings to cause a variety of nosocomial infections, including those found in the blood, respiratory tract, urinary tract, and on the skin. Hospitalized patients, especially those with immunocompromising conditions or implanted medical devices, are at increased risk of significant morbidity and mortality. The aim of this study was to better understand the clinical and demographic characteristics of the 13 case-patients identified during this investigation. **Methods:** A retrospective case series was conducted by reviewing medical records for case-patients with culture-confirmed S. maltophilia admitted between July and November. The CDC Healthcare-Associated Infection Outbreak Investigation Abstraction Form was used to systematically collect details about each case-patient’s hospitalization and course of illness. A Gantt chart was developed in Microsoft Excel to illustrate key events during their hospitalization. **Results:** Of the 13 case-patients, 69% were male and the median age was 69 years (range: 30 to 77). All S. maltophilia infections were hospital-acquired (>3 days after admission) with 92% being respiratory and 46% resistant to more than one class of antibiotics. All case-patients were admitted to the ICU and had known risk factors associated with developing S. maltophilia infection, including intubation (100%) and receiving antibiotic therapy prior to infection (77%). Other major risk factors included invasive surgery (77%), co-infections (77%), chronic respiratory disease (62%), hypertension (54%), and renal failure (31%). All were severely immunocompromised. Forty-six percent of the case-patients died from complications associated with their illness. **Conclusion:** This is the first S. maltophilia outbreak reported in Alabama. The findings of this case series underscored the importance of employing strict infection prevention measures to reduce poor health outcomes and how strong antibiotic stewardship programs are needed to limit transmission among vulnerable patient populations in these settings. It is recommended that hospitals conduct routine environmental sampling and have a WMP that is effective in limiting S. maltophilia.

